# Livestock-Associated Methicillin and Multidrug Resistant *Staphylococcus aureus* Is Present among Industrial, Not Antibiotic-Free Livestock Operation Workers in North Carolina

**DOI:** 10.1371/journal.pone.0067641

**Published:** 2013-07-02

**Authors:** Jessica L. Rinsky, Maya Nadimpalli, Steve Wing, Devon Hall, Dothula Baron, Lance B. Price, Jesper Larsen, Marc Stegger, Jill Stewart, Christopher D. Heaney

**Affiliations:** 1 Department of Epidemiology, University of North Carolina, Chapel Hill, North Carolina, United States of America; 2 Department of Environmental Sciences and Engineering, University of North Carolina, Chapel Hill, North Carolina, United States of America; 3 Rural Empowerment Association for Community Help (REACH), Warsaw, North Carolina, United States of America; 4 Department of Environmental and Occupational Health, George Washington University, Washington, District of Columbia, United States of America; 5 Microbiology and Infection Control, Statens Serum Institut, Copenhagen, Denmark; 6 Department of Environmental Health Sciences, Bloomberg School of Public Health, Johns Hopkins University, Baltimore, Maryland, United States of America; 7 Department of Epidemiology, Bloomberg School of Public Health, Johns Hopkins University, Baltimore, Maryland, United States of America; Institut National de la Recherche Agronomique, France

## Abstract

**Objectives:**

Administration of antibiotics to food animals may select for drug-resistant pathogens of clinical significance, such as methicillin-resistant *Staphylococcus aureus* (MRSA). In the United States, studies have examined prevalence of MRSA carriage among individuals exposed to livestock, but prevalence of multidrug-resistant *S. aureus* (MDRSA) carriage and the association with livestock raised with versus without antibiotic selective pressure remains unclear. We aimed to examine prevalence, antibiotic susceptibility, and molecular characteristics of *S. aureus* among industrial livestock operation (ILO) and antibiotic-free livestock operation (AFLO) workers and household members in North Carolina.

**Methods:**

Participants in this cross-sectional study were interviewed and provided a nasal swab for *S. aureus* analysis. Resulting *S. aureus* isolates were assessed for antibiotic susceptibility, multi-locus sequence type, and absence of the *scn* gene (a marker of livestock association).

**Results:**

Among 99 ILO and 105 AFLO participants, *S. aureus* nasal carriage prevalence was 41% and 40%, respectively. Among ILO and AFLO *S. aureus* carriers, MRSA was detected in 7% (3/41) and 7% (3/42), respectively. Thirty seven percent of 41 ILO versus 19% of 42 AFLO *S. aureus*-positive participants carried MDRSA. *S. aureus* clonal complex (CC) 398 was observed only among workers and predominated among ILO (13/34) compared with AFLO (1/35) *S. aureus-*positive workers. Only ILO workers carried *scn*-negative MRSA CC398 (2/34) and *scn*-negative MDRSA CC398 (6/34), and all of these isolates were tetracycline resistant.

**Conclusions:**

Despite similar *S. aureus* and MRSA prevalence among ILO and AFLO-exposed individuals, livestock-associated MRSA and MDRSA (tetracycline-resistant, CC398, *scn*-negative) were only present among ILO-exposed individuals. These findings support growing concern about antibiotics use and confinement in livestock production, raising questions about the potential for occupational exposure to an opportunistic and drug-resistant pathogen, which in other settings including hospitals and the community is of broad public health importance.

## Introduction

*Staphylococcus aureus* is an opportunistic pathogen carried by animals and approximately one third of humans, primarily in the nares [1]. *S. aureus* causes a range of human infections, from superficial to systemic [2] and strains resistant to methicillin (MRSA) have become leading causes of morbidity and mortality in the United States [3], [4]. In recent years, emergent multidrug-resistant strains of *S. aureus* (MDRSA) have made treatment of *S. aureus* infections more protracted, more burdensome, and less successful [5].

The epidemiology of *S. aureus* infection is evolving rapidly. Recognition of the prominence of community-acquired compared with hospital-acquired infections [6], the importance of MDRSA (that are not necessarily resistant to methicillin) as causes of infection [7], and the role of nasal carriage, which has been shown to increase the risk of subsequent *S. aureus* infection in clinical settings [8], is increasing. However, the origins and routes of transmission of community-acquired *S. aureus* infection as well as reservoirs of selective pressure that drive emergent strains and drug resistance remain poorly characterized in the United States [9].

Industrial food-animal production facilities have been identified as a source of human exposure to antibiotic-resistant *S. aureus*, including MRSA and MDRSA [10]. The practices of intensive confinement and administration of antibiotics to animals – including non-therapeutically for growth promotion – are commonly used in industrial food-animal production and provide a reservoir for the selection of novel, antibiotic-resistant bacteria that can be exchanged between animals and humans.

Concerns have been rising about the dissemination of clonal complexes (CCs) of *S. aureus* that circulate among livestock and are now recognized as causes of human infections in the community and hospitals [11]. Of particular interest is *S. aureus* CC398, first reported among pig farmers in France in 2005 [12] and later among pigs and pig handlers across Europe [13] and North America [14]. Although human infections from livestock-associated *S. aureus* CC398 and MRSA CC398 appear to be rare at this time in the United States [13], spread of MRSA CC398 from livestock to humans has increased the burden of MRSA infections in many European countries, including Denmark, Germany, and the Netherlands [13], [15]–[18]. Despite a well-developed knowledge base in Europe [16]–[18], little is known about antibiotic-resistant *S. aureus* carriage among livestock workers and their household members in the United States, particularly individuals exposed to livestock that are not raised using antibiotic inputs [19].

In addition, few studies examining carriage of *S. aureus* among individuals in contact with livestock have attempted to differentiate human- from livestock-associated strains based on phenotypic and molecular markers. While some have defined livestock-associated *S. aureus* based on resistance pattern and CC [20], [21], few have incorporated recent evidence suggesting that absence of the bacteriophage-encoded *scn* immune evasion cluster (IEC) gene may be a useful indicator of *S. aureus* livestock adaptation [20], [22], [23]. *scn* has been detected at a relatively low frequency among diverse collections of *S. aureus* isolates obtained from livestock (2%–35%) compared to those obtained from humans (90–100%) [20], [22], [23]. Loss of the IEC cluster has been observed during two independent human-to-animal host jumps by *S*. *aureus* ST5 and CC398, suggesting that the *scn* gene is selected against in animal hosts [20], [24]. Thus, among food animal production workers, the *scn* gene may be useful to differentiate livestock-to-human transmission from community-acquired human colonization and help improve assessment of occupational exposure risks associated with livestock production.

In the present study, we aimed to examine the prevalence of carriage and phenotypic and molecular characteristics of *S. aureus* among industrial and antibiotic-free livestock workers and their household members in North Carolina. We also investigated potential occupational risk factors for carriage and evidence of potential strain concordance within households between workers and household members.

## Methods

### Ethics Statement

The UNC Public Health-Nursing institutional review board approved this study. Before participating, adult participants provided written informed consent. Written parental permission and informed assent were collected for participants seven to 17 years of age.

Data were collected between May and December 2011 in North Carolina, USA by researchers from the University of North Carolina at Chapel Hill (UNC) with organizers from two community organizations – the Rural Empowerment Association for Community Help (REACH) and the North Carolina Environmental Justice Network (NCEJN).

### Study Population

Participants were enrolled using a snowball sampling approach. Individuals were eligible if they worked at an operation that housed swine or poultry, resided in North Carolina, were at least 18 years old, and could understand English or Spanish. Up to two individuals living in the same household as a participating worker were eligible to participate if they were at least seven years old and able to understand English or Spanish.

Participants reported occupational activities, contact with livestock, household and environmental exposures, personal activities, medical history, and demographic information. Participants who worked at operations where animals were raised exclusively in confinement were classified as industrial livestock operation (ILO) workers. Those who reported working at operations that raised animals outdoors, on pasture, and did not administer antibiotics to raise animals were classified as antibiotic-free livestock operation (AFLO) workers. Each worker’s respective household members were included in the same group as the worker, thereby defining two exposure groups –1) ILO workers and their household members; and, 2) AFLO workers and their household members. Because of concerns about privacy and confidentiality with respect to employment among ILO workers where there is a history of intimidation tactics [25], no identifying information about livestock operations was collected – including name of livestock operation of employment and whether participants worked at the same livestock operation as other participants.

### Detection of *S. aureus* and MRSA

Study personnel obtained a nasal swab from both nares of each participant using a BD BBL™ CultureSwab™. Swabs were inoculated into 10 ml of Mueller-Hinton broth containing 6.5% NaCl, then incubated overnight at 37°C. To isolate presumptive *S. aureus*, a loopful of Mueller-Hinton broth was streaked onto CHROMagar™ Staph aureus plates (BD, Franklin Lakes, NJ) and incubated at 37°C for 24 hours. Colonies with morphology characteristic of *S. aureus* were streaked to isolation. To isolate presumptive MRSA, 1 ml of bacteria-enriched Mueller-Hinton broth was subsequently transferred to 10 ml of tryptic soy broth containing 2.5% NaCl, 3.5 mg/L cefoxitin, and 10 mg/L aztreonam, then incubated overnight at 37°C. Isolation of presumptive MRSA colonies from tryptic soy broth followed the same procedure as for presumptive *S. aureus*.

DNA from presumptive *S. aureus* and MRSA colonies was extracted using Qiagen’s DNeasy Blood & Tissue kit (Qiagen, Valencia, CA), with a protocol for gram-positive bacteria recommended by the manufacturer. A multiplex PCR assay was used to amplify the 16S rRNA, *nuc*, and *mecA* genes in all extracted isolates [26]. Amplified gene products were confirmed by gel electrophoresis. Isolates that were positive for the 16S rRNA and *nuc* genes were classified as *S. aureus*. *S. aureus* isolates that were positive for the *mec*A gene were classified as MRSA. Molecular findings were confirmed using catalase and tube coagulase testing with rabbit plasma (BD BBL, Franklin Lakes, NJ).

### Antibiotic Susceptibility

One isolate per *S. aureus*-positive individual was assessed for susceptibility to a panel of 12 antibiotic classes, comprising 16 antibiotics (see **Supporting Information Table S1 in File S1** for listing of antibiotics). The Kirby-Bauer disk diffusion method was used to assess susceptibility to all antibiotics except vancomycin; diameter interpretations were based on Clinical and Laboratory Standards Institute (CLSI) guidelines [27]. Inducible clindamycin resistance was evaluated in erythromycin-resistant isolates using the D-zone test [28]. Vancomycin susceptibility was assessed for all isolates on brain heart infusion agar supplemented with 5 mg/L vancomycin hydrochloride using methods adapted from CLSI [29]. MRSA isolates were verified phenotypically by complete resistance to oxacillin and complete or intermediate resistance to ceftriaxone. Isolates that demonstrated complete resistance to three or more classes of antibiotics were classified as MDRSA [30]. MRSA isolates meeting the definition of MDRSA were classified as multidrug-resistant MRSA.

### Whole-genome Sequencing and Molecular Typing

Whole-genome sequencing of one isolate per *S. aureus*-positive individual was carried out on an Illumina Genome Analyzer*_IIx_* at the Translational Genomics Research Institute (TGen) (Flagstaff, AZ), as described elsewhere [20]. *De novo* assemblies were generated by Velvet [31]. Multi-locus sequence typing (MLST) was performed on *de novo* assemblies as described elsewhere [32], and CCs were determined using eBURST (version 3; http://eburst.mlst.net) and the stringent group definition (6/7 shared alleles) [33]. *S. aureus* isolates were assigned to standardized sequence types using open-access, online tools (www.mlst.net). Isolates observed to have novel alleles or allelic profiles by in-silico typing were confirmed by PCR and Sanger sequencing [34], then submitted to the MLST database. *De novo* assemblies were analyzed by BLASTN using the *scn* gene (GenBank accession no. NC_009641, NWMN_1876) as query.

### Statistical Analysis

We compared percentages of participants with potential demographic and environmental risk factors for carriage in the two exposure groups. We also compared workers’ reported direct contact with animals on an average day and the hours per week spent in direct contact with animals at work. Chi-square values and degrees of freedom are reported from Pearson’s chi-square tests for independence. We do not report corresponding p-values because they are often interpreted incorrectly in non-randomized studies as reflecting the probability that the results would be observed by chance under the null hypothesis that no difference exists between the groups being compared [35].

We calculated the prevalence of nasal carriage of *S. aureus* in both groups. Among participants carrying *S. aureus*, we calculated the proportion resistant to the 16 antibiotics and the proportion of MRSA, MDRSA, *S. aureus* CC398, and *S. aureus* lacking the *scn* gene. Where sample size allowed, we examined associations between the demographic and environmental risk factors and carriage of *S. aureus,* and the prevalence of the aforementioned phenotypic and molecular characteristics.

Crude prevalence ratios (PR) comparing carriage among the ILO group to carriage among the AFLO group along with 95% confidence intervals (CI) were generated from log binomial models using generalized estimating equations with an unstructured correlation matrix to account for the non-independence of observations within households [36]. Analyses were conducted separately for workers; however, workers and household members were also evaluated together because of their shared environments. Adjusted PRs are not presented because key variables were distributed differently between the groups. We report 95% CIs to provide information regarding the precision of the observed prevalence ratios.

To identify the proportion of *S. aureus* potentially associated with livestock contact, we considered the distribution of three indicators of livestock association – resistance to tetracycline, presence of CC398, and absence of the *scn*-gene – within the ILO and AFLO groups. We refer to isolates with all three of these markers as livestock-associated *S. aureus*.

Among workers, we examined associations between work activities that involve direct contact with animals and the prevalence of MDRSA and *S. aureus* exhibiting indicators of livestock-association.

Finally, we examined strain concordance using phenotypic and molecular characteristics of *S. aureus* isolated from participants living in the same household. All statistical analyses were performed using SAS 9.2 (SAS Institute Inc., Cary, NC).

## Results

### Participant Characteristics

Participant characteristics are presented in **Table 1**. Among 99 ILO and 105 AFLO participants, 80.8% and 87.6%, respectively, were workers. Most of the AFLO group identified as non-Hispanic white (79.1%), and most of the ILO group identified as Hispanic (81.8%). AFLO participants reported higher levels of education and a greater proportion reported pets living in the home than ILO participants.

**Table 1:** Distribution of characteristics among all study participants by exposure group, North Carolina, 2011.

		ILO	AFLO	
		N = 99	N = 105	Chi-square
		%	%	value (df)^a^
**Participant Type**				3.24 (2)
	Worker	80.8	87.6	
	Adult household member	10.1	3.8	
	Minor household member	9.1	8.6	
**Age**				7.62 (4)
	<18	9.1	9.5	
	18–24	12.1	25.7	
	25–34	28.3	27.6	
	35–44	22.2	13.3	
	≥45	28.3	23.8	
**Gender**				0.24 (1)
	Male	59.6	56.2	
	Female	40.4	43.8	
**Race/Ethnicity**				126.13 (2)
	Black, non-Hispanic	16.2	2.9	
	White, non-Hispanic	2.0	79.1	
	Hispanic	81.8	17.1	
**Education** a				33.75 (1)
	< High School	65.4	17.7	
	≥ High School	34.6	82.4	
**Antibiotic Use**				0.34 (1)
	≥3 months ago	85.6	88.4	
	<3 months ago	14.4	11.7	
**Hospitalization**				0.27 (1)
	≥3 months ago	97.0	98.1	
	<3 months ago	3.0	1.9	
**Pets inside home**				35.32 (1)
	No	91.8	54.3	
	Yes	8.3	45.7	
**Contact sports**				2.55 (1)
	≥3 months ago	82.5	73.1	
	<3 months ago	17.5	26.9	

ILO = industrial livestock operation.

AFLO = antibiotic-free livestock operation.

a Restricted to participants ≥25 years old.

**Table 2** illustrates work exposures related to direct contact with animals for ILO and AFLO workers. ILO workers mainly reported direct contact with either swine or poultry, although one worker reported direct contact with both. Most AFLO workers reported direct contact with both swine and poultry and 79.3% reported direct contact with other livestock as compared to only 2.5% of ILO workers. ILO workers reported direct contact with larger numbers of animals and for more hours/week than AFLO workers. Most AFLO household members reported direct contact with livestock at home while few ILO household members reported direct contact with livestock (information not shown).

**Table 2:** Characteristics of livestock operations and worker exposures, North Carolina, 2011.

		ILO		AFLO		Chi-square
		N = 80	%	N = 92	%	value (df)
**Direct contact with livestock**		77	96.3	92	100.0	3.51 (1)
**Direct contact with pigs** a		59	73.8	62	67.4	1.76 (1)
**Direct contact with poultry** a		19	23.8	65	70.7	35.44 (1)
**Direct contact with other animals**		2	2.5	73	79.3	100.03 (1)
**Number of pigs in direct contact with/day** b						108.16 (4)
None		21	26.3	30	32.6	
1–249		1	1.3	53	57.6	
250–499		1	1.3	5	5.4	
500–999		3	3.8	4	4.3	
≥1000		54	67.5	0	0	
**Number of poultry in direct contact with/day** c						76.21 (4)
None		61	76.3	27	29.3	
1–249		0	0	52	56.5	
250–499		0	0	5	5.4	
500–999		0	0	1	1.1	
≥1000		19	23.8	7	7.6	
**Hours/week worked in direct contact with livestock**						132.02 (3)
None		3	3.8	0	0	
1–20 hours		5	6.3	81	88.0	
21–40 hours		10	12.5	11	12.0	
>40 hours^d^		62	77.5	0	0	

ILO = industrial livestock operation.

AFLO = antibiotic-free livestock operation.

a Categories are not exclusive.

b Restricted to workers reporting direct contact with pigs.

c Restricted to workers reporting direct contact with poultry.

d The maximum number of hours/week worked in direct contact with livestock was 73.5 and 35 hours for ILO and AFLO workers, respectively.

### Prevalence of *S. aureus*, MRSA, and MDRSA

**Figure 1:**
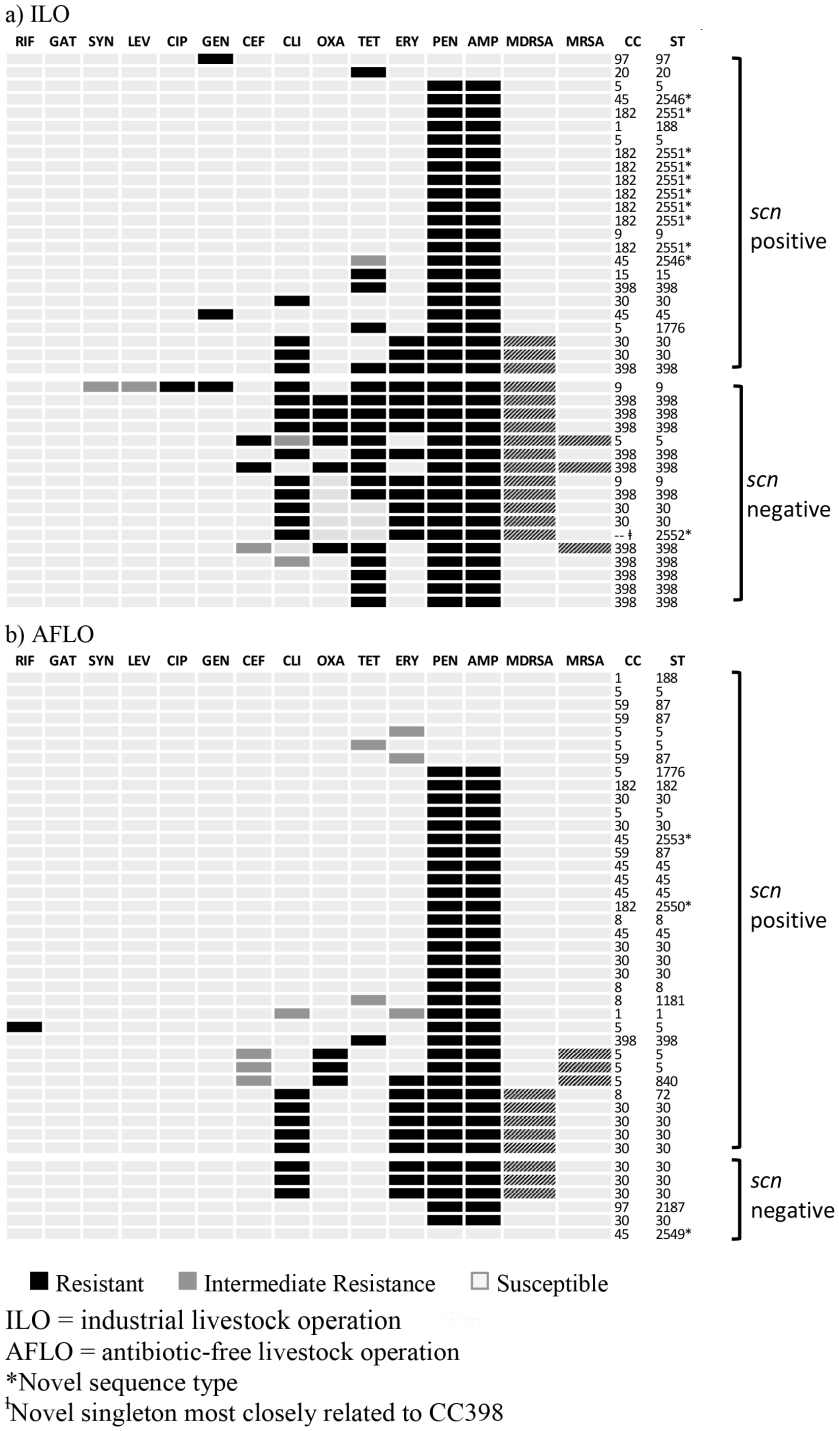
Antibiotic resistance profiles, clonal complexes, and multi-locus sequence types of all *S. aureus* isolates from (A) ILO and (B) AFLO participants, stratified by *scn* status, North Carolina, 2011.

Forty-one of 99 (41.4%) ILO and 42/105 (40.0%) AFLO participants carried *S. aureus* **(** **Table 3** **)**. Characteristics of all *S. aureus* isolates are described in **Figure 1**. MRSA was detected in 3/41 (7.3%) and 3/42 (7.1%) *S. aureus*-positive ILO and AFLO participants, respectively. Among *S. aureus*-positive participants, 15/41 (36.6%) ILO participants and 8/42 (19.0%) AFLO participants carried MDRSA. Two of three MRSA isolates from ILO participants were also multidrug-resistant (**Figure 1**). Multidrug-resistant MRSA was not detected among AFLO participants.

**Table 3:** Phenotypic and molecular characteristics of *S. aureus* carriage among study participants by livestock exposure group, North Carolina, 2011.

	ILO		AFLO		Prevalence Ratio
	No. positive/	%	No. positive/	%	(95% CI)^a^
	Total		Total		
***S. aureus***	41/99	41.4	42/105	40.0	1.0 (0.7, 1.5)
Worker	34/80	42.5	35/92	38.0	1.1 (0.8, 1.7)
Household Member	7/19	36.8	7/13	53.8	
**MRSA**	3/41	7.3	3/42	7.1	1.0 (0.2, 6.0)
Worker	3/34	8.8	3/35	8.6	1.0 (0.2, 5.9)
Household Member	0/7	0.0	0/7	0.0	
**MDRSA**	15/41	36.6	8/42	19.0	1.9 (0.9, 4.0)
Worker	13/34	38.2	8/35	22.9	1.7 (0.8, 3.5)
Household Member	2/7	28.6	0/7	0.0	
**Tetracycline-resistant ** ***S. aureus***	19/41	46.3	1/42	2.4	19.5 (2.7, 140)
Worker	19/34	55.9	1/35	2.9	19.6 (2.7, 140)
Household Member	0/7	0.0	0/7	0.0	
***S. aureus*** ** CC398**	13/41	31.7	1/42	2.4	13.3 (1.8, 98.7)
Worker	13/34	38.2	1/35	2.9	13.4 (1.8, 98.0)
Household Member	0/7	0.0	0/7	0.0	
***scn-*** **negative ** ***S. aureus***	17/41	41.5	6/42	14.3	2.9 (1.3, 6.4)
Worker	15/34	44.1	6/35	17.1	2.6 (1.2, 5.7)
Adult Household Member	2/7	28.6	0/7	0.0	
***scn-*** **negative, tetracycline-resistant ** ***S. aureus*** ** CC398**	11/41	26.8	0/42	0.0	– b
Worker	11/34	32.3	0/35	0.0	– b
Household Member	0/7	0.0	0/7	0.0	
***scn–*** **negative MRSA CC398**	2/41	4.9	0/42	0.0	– b
Worker	2/34	5.9	0/35	0.0	– b
Household Member	0/7	0.0	0/7	0.0	
***scn-*** **negative MDRSA CC398**	6/41	14.6	0/42	0.0	– b
Worker	6/34	17.6	0/35	0.0	– b
Household Member	0/7	0.0	0/7	0.0	

ILO = industrial livestock operation.

AFLO = antibiotic-free livestock operation.

PR = prevalence ratio.

a PR (95% CI) comparing carriage among ILO participants to AFLO participants.

b No estimate of the PR (95% CI) is provided due to no occurrence of the outcome of interest in at least one group.

Participant characteristics shown in **Table 1** showed little association with carriage of *S. aureus* or MDRSA within groups (information not shown). Examination of associations between these characteristics and MRSA carriage was not possible due to small numbers.

### Antibiotic Drug Resistance Phenotypes

Of the 16 antibiotics tested, complete or intermediate resistance was observed to all antibiotics except vancomycin, linezolid, and sulfamethoxazole/trimethoprim (**Figure 1**). Compared to *S. aureus*-positive AFLO participants 1/42 (2.4%), complete resistance to tetracycline was present in 19/41 (46.3%) *S. aureus*-positive ILO participants (Prevalence Ratio [PR]: 19.5; 95% confidence interval [CI]: 2.7, 140.4) (**Table 3**).

### MLST of S. aureus

MLST revealed a diversity of CCs, or clusters of closely-related sequence types, among individuals with *S. aureus*, including CC1, CC5, CC8, CC9, CC30, CC45, CC97, and CC398 (**Figure 1**). Thirteen of 41 (31.7%) *S. aureus*-positive ILO participants carried CC398, compared with 1/42 (2.4%) *S. aureus*-positive AFLO participants (PR: 13.3; 95% CI: 1.8, 98.7) (**Table 3**). All ILO or AFLO participants who carried *S. aureus* CC398 were workers. Six new sequence types were detected in this study, and were distributed primarily within the ILO group **(** **Figure 1** **)**. A novel singleton (ST2552) most closely related to CC398– a double locus variant (sharing 5/7 alleles with the founder of this CC) – was detected in the ILO group **(** **Figure 1** **)**.

### Prevalence of *scn-*negative *S. aureus*

*scn*-negative *S. aureus* isolates were more prevalent among ILO (41.5%; 17/41) than AFLO (14.3%; 6/42) participants (PR: 2.9; 95% CI: 1.3, 6.4) (**Table 3**). All MRSA isolates detected among ILO participants (3/3) were *scn*-negative, whereas none of the MRSA isolates from AFLO participants were *scn*-negative (**Figure 1**). Among ILO participants, 70.6% (12/17) of *scn*-negative isolates compared with 12.5% (3/24) of *scn*-positive isolates were MDRSA (**Figure 1**).

### Markers of Livestock Association

Overlap between phenotypic (tetracycline resistance) and molecular (CC398, *scn*-negative) markers of livestock association (**Figure 1**) was predominantly observed among ILO participants. Among ILO participants who carried *scn*-negative *S. aureus*, 82.3% (14/17) exhibited at least one other characteristic of livestock association (CC398 or tetracycline resistance) and 64.7% (11/17) exhibited all three characteristics. *S. aureus* CC398 isolates were predominantly tetracycline-resistant, *scn-*negative, and observed among ILO participants. The single *S. aureus* CC398 isolate observed among AFLO participants was tetracycline resistant but *scn*-positive. *scn*-negative *S. aureus* CC398, *scn*-negative MRSA CC398, and *scn*-negative MDRSA CC398 were only observed among ILO participants **(** **Table 3** **)**. Three of six *scn-*negative *S. aureus* isolates among AFLO participants were MDRSA (**Figure 1**), but no overlap in the three characteristics of livestock association was observed among AFLO participants.

### Occupational and Environmental Risk Factors

Associations between reported work activities and carriage of MDRSA and *S. aureus* displaying phenotypic and molecular markers of livestock association are shown in **Table 4**. ILO workers with direct contact with breeding pigs demonstrated a greater prevalence of MDRSA nasal carriage (PR: 2.4; 95% CI: 1.0, 5.7) and tetracycline-resistant *scn-*negative *S. aureus* CC398 (PR: 2.5; 95% CI: 1.0, 6.4), compared with workers not working in direct contact with breeding pigs.

**Table 4:** Associations between reported work activities and carriage of MDRSA and tetracycline-resistant *scn*-negative *S. aureus* CC398 among ILO workers^a^ carrying *S. aureus*.

		Total	MDRSA			Tetracycline-resistant *scn*-negative *S. aureus* CC398 b		
		N = 34	N = 13	Prevalence	PR (95% CI)	N = 11	Prevalence	PR (95% CI)
**Direct contact with pigs**								
	No	6	2	33.3	Ref	1	16.7	Ref
	Yes	28	11	39.3	1.2 (0.3, 4.4)	10	35.7	2.1 (0.3, 14.4)
**Direct contact with poultry**								
	No	28	11	39.3	Ref	10	35.7	Ref
	Yes	6	2	33.3	0.8 (0.2, 3.1)	1	16.7	0.5 (0.1, 3.1)
**Were cut/scratched while in direct contact with livestock**								
	No	22	9	40.9	Ref	8	36.4	Ref
	Yes	12	4	33.3	0.8 (0.3, 2.0)	3	25.0	0.7 (0.2, 2.2)
**Draw blood/collect fluids from pigs**								
	No	27	8	29.6	Ref	10	37.0	Ref
	Yes	7	5	71.4	2.4 (1.1, 5.1)	1	14.3	0.4 (0.1, 1.9)
**Work with breeding pigs**								
	No	23	6	26.1	Ref	5	21.7	Ref
	Yes	11	7	63.6	2.4 (1.0, 5.7)	6	54.5	2.5 (1.0, 6.4)
**Handle livestock that were dead**								
	No	5	2	40	Ref	3	60.0	Ref
	Yes	29	11	37.9	0.9 (0.3, 3.1)	8	27.6	0.5 (0.2, 1.1)

ILO = industrial livestock operation.

PR = Prevalence ratio.

a Associations among AFLO workers are not presented due to small numbers of reported activities in the group who carried *S. aureus*.

b Due to overlap among tetracycline-resistance, *scn-*negative, and CC398 isolates and similarity of estimates, we present prevalence ratios and 95% confidence intervals for tetracycline-resistant *scn*-negative *S. aureus* CC398.

Results regarding the concordance of *S. aureus* found among workers and their respective household members are presented in **Supporting Information Table S2a–b in File S1**. There were few similarities in *S. aureus* phenotype or molecular characteristics within households.

## Discussion

Despite a similar prevalence of carriage of *S. aureus* and MRSA among ILO and AFLO-exposed individuals, the distribution of *S. aureus* demonstrating characteristics of livestock association (tetracycline resistance, CC398, *scn*-negative) differed between groups. This is the first report of a livestock-associated strain of *S. aureus* CC398 (including MRSA CC398 and MDRSA CC398) in North Carolina. We observed that individuals who worked with livestock raised using intensive confinement and antibiotic inputs, including antibiotics administered non-therapeutically, carried MRSA and MDRSA with multiple markers of livestock-association (tetracycline-resistance, CC398, *scn-*negative). Individuals who worked with livestock raised without the use of antibiotics and confinement were not observed to be carrying MRSA and MDRSA demonstrating multiple markers of livestock-association. We also observed some indication that, regardless of markers of livestock association, there was an elevated proportion of *S. aureus* that was MDRSA among ILO compared to AFLO participants.

Previous work demonstrates the potential for transmission of *S. aureus* between animals and humans on ILO and AFLO operations [37]–[40]. A study conducted on Dutch dairy farms [37] and another across livestock production sectors in Belgium [38] observed nearly indistinguishable MRSA CC398 isolates in humans and various types of animals on the same property. Both of these studies focused on MRSA CC398, but also observed transmission of multidrug-resistant MRSA CC398. In the present study, we observed a higher prevalence of livestock-associated *S. aureus* strains among ILO participants and a higher prevalence of human epidemic clones of *S. aureus* among AFLO participants. This is consistent with previously published studies of *S. aureus* carriage among workers and pigs at conventional [14] and non-conventional [19] farms in the United States. However, since we studied human carriage only, our ability to draw inferences about the directionality, frequency, or intensity of *S. aureus* exchange between animals and humans in ILO or AFLO settings is limited.

*S. aureus* CC398, which has been identified as an emerging livestock-associated *S. aureus* strain in Europe [41]–[43], is commonly considered as a molecular marker of livestock-associated *S. aureus*. Near universal resistance to tetracycline has been reported among *S. aureus* CC398 isolates from livestock and humans in close contact with livestock [20], [41] and tetracycline-susceptibility has been reported among *S. aureus* CC398 collected from humans with no known livestock exposure [21], [44]. These observations are consistent with findings reported by Price et al. (2012) that CC398 acquired resistance to methicillin and tetracycline after the introduction to livestock from humans [20]. In the Price et al. (2012) study of historical isolates, the tetracycline resistance gene *tet*(M) was found among 99% of isolates from livestock and absent from isolates obtained from humans [20]. Thus, tetracycline resistance in conjunction with CC398 has been used as a marker of livestock-associated *S. aureus.* Since the 1950s, tetracycline and its derivatives have been used extensively in US livestock production for non-therapeutic purposes such as growth promotion [45], [46]. In 2011, approximately 5.6 million pounds of tetracycline were sold and distributed in the United States, the greatest amount for any drug class approved for use in food-producing animals [47]. Our observation of a greater prevalence of tetracycline-resistant *S. aureus,* including MRSA and MDRSA, among ILO compared with AFLO participants is consistent with the historical and ongoing use of tetracycline in industrial livestock production. Although tetracycline resistance is important to note with regard to its use in animal production, it is also used in clinical settings to treat human infections, including skin and soft tissue *S. aureus* infections [48].

Recent work has demonstrated that absence of the *scn* gene may aid in the differentiation of animal origins of *S. aureus* carried by humans exposed to livestock [20], [22]–[24]. In the present study, the majority of *scn-*negative strains carried by ILO participants (70.6%), belonged to CC398. All other *scn*-negative strains carried by participants also belonged to CCs that have previously been described in animals (CC97, CC45, CC30, CC9, CC8, CC5, CC1) [49], [50]. CC30 has recently been described in livestock in Europe [50] and was the most prevalent *scn*-negative strain among AFLO participants in our study. The majority of *scn*-negative isolates from ILO participants (82.3%) were resistant to tetracycline while those from AFLO participants were not. Within the ILO group, substantial overlap occurred among markers of livestock-association (tetracycline resistance, CC398, *scn*-negative). Within the AFLO group, little overlap of the markers of livestock-association was observed. One CC398 isolate was observed among the AFLO group, and while it demonstrated tetracycline resistance, it carried the *scn* gene. Further, among the AFLO participants no *scn-*negative isolates were tetracycline resistant or CC398. These findings suggest that while both groups may be exposed to MRSA and MDRSA, individuals working in the ILO setting may be exposed to a larger or more sustained livestock reservoir of MRSA and MDRSA, or that these livestock-associated MRSA and MDRSA are transmitted more frequently between animals and humans in the ILO as compared to the AFLO setting.

Although *scn* appears to be a promising marker, additional work is needed to establish its validity as an indicator of livestock-associated *S. aureus.* It is not known how long *scn* is harbored by human-origin *S. aureus* strains once passed to livestock hosts, nor are the factors that might influence the speed of *scn* bacteriophage loss fully understood, although previous work showed that feed-delivered antibiotics can induce prophage loss [51]. Establishing the temporal dynamics of *scn* presence/absence among *S. aureus* passed between humans and animals will help determine its utility as a marker of livestock association.

When using all participants as the denominator – to facilitate population prevalence comparisons with other studies – the overall prevalence of *S. aureus* (39%) and MRSA (3.4%) in our study were slightly greater than estimates of prevalence of *S. aureus* (30.4%; 95% CI: 29.4, 31.5%) and MRSA (1.2%; 95% CI: 0.9, 1.5%) carriage in the general US population from 2001–2004 [1]. Additional comparison of *S. aureus* and MRSA nasal carriage prevalence with populations similar to the present study is challenging as few other studies examine carriage among populations not receiving or working in medical care. There is little basis for making comparisons to other MDRSA population prevalence estimates because most other studies investigate MRSA only, or do not provide sufficient information to interpret study population denominators for MDRSA carriage.

Smith et al. examined MRSA carriage among 20 workers employed at two industrial swine production facilities in Iowa and Illinois [14]. MRSA was detected among swine in one facility where 9/14 workers were also found to carry MRSA. All observed MRSA was tetracycline-resistant CC398. No MRSA was detected among swine or workers in the second facility, leading to an overall MRSA carriage prevalence of 45% among workers. Although the overall MRSA prevalence among ILO workers reported in the present study (3/80; 4%) was lower than that reported by Smith et al., the present study included workers from a larger number and a more diverse cross-section of ILOs. While we did not ask workers to reveal their place of employment for reasons of privacy and confidentiality, our sampling of ILO workers from a larger number of operations is based in part on knowledge of the average number of employees at each operation. Data from the 2007 Census of Agriculture conducted by the United States Department of Agriculture indicate that the reported industry-wide average number of employees on hog and pig farming operations in North Carolina was 6.8 among 938 out of 1619 operations that reported having hired labor (the remaining 681 operations reported having no hired labor) [52].

Smith et al. (2013) also examined carriage of MRSA among weaned pigs and 148 workers on ILOs and AFLOs in Iowa, Illinois, Minnesota, Ohio, and North Carolina [40]. MRSA-positive herds were only found in Iowa and Illinois. MRSA was not found among AFLO pigs or among AFLO workers, whereas we found 3 of 92 AFLO workers (3.3%) to be MRSA-positive. Smith et al. (2013) observed 8.5% MRSA prevalence among ILO pigs. Overall, 31 ILO workers were positive for MRSA carriage and 27 of 31 (87%) MRSA-positive workers were employed at ILOs where MRSA was detected among pigs. Further comparison with our study’s findings is not possible because ILO and AFLO worker denominator information was not included in Smith et al. (2013) [40].

The present study would be strengthened by sampling animals and production facilities [14], [53]; however, we did not have access during the course of the study. Access to livestock operations by the public health research community in Europe and other countries has allowed a robust body of evidence to develop there regarding the extent of exposure to *S. aureus*, MRSA, and MDRSA among ILO workers as well as the potential dissemination of livestock-associated strains into the broader community. Though our study may be viewed as small, to the best of our knowledge, it is one of the largest studies on this topic in the United States to date. Furthermore, it was not restricted to ILOs that have agreed to participate in research.

In principle, the difference in characteristics of *S. aureus* observed among the ILO compared to the AFLO group could be related to livestock exposures, to other differences between the AFLO and ILO participant populations, or to a combination of the two. The ILO and AFLO study populations appeared different in their demographic make-up; 82% of ILO participants in our study self-identified as Hispanic whereas 79% of AFLO participants identified as non-Hispanic white. Industrial swine operations in North Carolina are disproportionately located in low income communities of color in the eastern portion of the state [54]. Differences in ethnicity and education between ILO and AFLO workers reflect differences in the communities where they live, and differences in *S. aureus* subtypes circulating in these populations could affect differences in carriage observed in this study. Although some factors (e.g. recent antibiotic use and hospitalization) associated with risk of carriage of antibiotic-resistant *S. aureus* were similar between groups or greater among the AFLO group (pets in the home) [55]–[57]; the latter would tend to offset any differences in the study groups due to occupational exposures.

Because we did not sample from an enumerated base population (e.g., employee rosters) it is unclear how generalizable these results may be to all ILO workers in North Carolina or the United States. Notwithstanding this limitation, our findings raise important questions about the frequency of potential occupational exposure to antibiotic-resistant *S. aureus* among an estimated 292,000 livestock workers in the United States in 2012 [52], [58], [59].

Despite current understanding of livestock-associated MRSA as a relatively rare cause of human infection in the United States (which may be limited due to a lack of systematic national surveillance) [13], there is public health concern about potential broad dissemination of drug-resistant *S. aureus* to the general public. Although others have observed evidence of phenotypic and molecular strain concordance within households [60], we did not observe *S. aureus* strain concordance between workers and household members. However, relatively few household members participated in this study and consequently this finding should be interpreted with caution. We also assessed only one *S. aureus* colony per colonized individual for all outcomes presented. It is possible that some individuals carried multiple *S. aureus* strains. We may not have captured all carriage states of some individuals and consequently may have missed potential occurrences of strain concordance within households. To advance understanding of patterns of potential household transmission, future studies should address these limitations.

The results reported here show that the proportion of *S. aureus* identified as MDRSA and the proportion exhibiting phenotypic and molecular markers of livestock association was elevated among individuals exposed to the ILO environment compared to those exposed to the AFLO environment. Carriage of *scn*-negative MRSA CC398 and *scn*-negative MDRSA CC398 was limited to individuals with direct exposure to ILO production – all of these isolates were also resistant to tetracycline. Whether or not these livestock-associated *S. aureus* strains (including MRSA and MDRSA) pose a health risk to workers and the broader public requires further investigation. Overall, our findings support growing concern about antibiotic use and confinement in livestock production, and raise questions about the potential for occupational exposure to an opportunistic and drug-resistant pathogen which in other settings including hospitals and the community is a major cause of morbidity and mortality in the United States [4], [61] and globally [6], [7].

## Supporting Information

File S1 Table S1: List of antibiotics by antibiotic class used in antibiotic-susceptibility testing. Table S2a–b: Phenotypic and molecular characteristics of *S. aureus* detected among households by exposure groups. Individuals in households shaded in gray displayed some level of strain concordance between worker and household member carriage status.
(DOCX)
