# Supplementary material for: Livestock-Associated Methicillin and Multidrug Resistant Staphylococcus aureus Is Present among Industrial, Not Antibiotic-Free Livestock Operation Workers in North Carolina
Source: PLoS One. 2013 Jul 2;8(7):e67641. doi: 10.1371/journal.pone.0067641 (PMC3699663; doi:10.1371/journal.pone.0067641)
Supplement: File S1 — Table S1: List of antibiotics by antibiotic class used in antibiotic-susceptibility testing. Table S2a–b: Phenotypic and molecular characteristics of S. aureus detected among households by exposure groups. Individuals in households shaded in gray displayed some level of strain concordance between worker and household member carriage status. (DOCX) [file pone.0067641.s001.docx]

**Supporting Information File S1**

**Livestock-associated methicillin and multidrug resistant *Staphylococcus aureus* is present among industrial, not antibiotic-free livestock operation workers in North Carolina**

**Table of Contents:**

|  |  |
| --- | --- |
| **Table S1.** List of antibiotics by antibiotic class used in antibiotic-susceptibility testing. | p. 3 |
| **Table S2a-b**. Phenotypic and molecular characteristics of *S. aureus* detected among households by exposure groups. Individuals in households shaded in gray displayed some level of strain concordance between worker and household member carriage status. | p. 4-6 |
|  |  |

**Table S1.** List of antibiotics by antibiotic class used in antibiotic-susceptibility testing.

| **Antibiotic class** | **Antibiotic tested** | **Concentration** |
| --- | --- | --- |
| aminoglycosides | gentamicin | 10 µg |
| β-lactams | ampicillin | 10 µg |
|  | oxacillin | 1 µg |
|  | penicillin | 10 units |
| cephalosporins | ceftriaxone | 30 µg |
| floroquinolones | ciprofloxacin | 5 µg |
|  | gatifloxacin | 5 µg |
|  | levofloxacin | 5 µg |
| glycopeptides | vancomycin | 5 µg/mL |
| lincosamides | clindamycin | 2 µg |
| macrolides | erythromycin | 15 µg |
| oxazolidones | linezolid | 30 µg |
| rifamycin | rifampin | 5 µg |
| streptogramins | quinupristin/dalfopristin | 15 µg |
| sulfonamide/  methoprim | sulfamethoxazole/trimethoprim | 23.75/1.25 µg |
| tetracycline | tetracycline | 30 µg |

**Table S2a-b.** Phenotypic and molecular characteristics of *S. aureus* detected among households by exposure groups. Individuals in households shaded in gray displayed some level of strain concordance between worker and household member carriage status.

1. ILO participants by household

| **Household** | **Participant Type** | ***S. aureus*** | **MRSA** | **MDRSA** | **Tetracycline- resistance** | ***scn*** | **CC**^b^ |
| --- | --- | --- | --- | --- | --- | --- | --- |
| 1 | Worker | Yes | No | Yes | Resistant | - | 398 |
|  | Worker | No |  |  |  |  |  |
| 4 | Worker | No |  |  |  |  |  |
|  | Household Member | Yes | No | No | Susceptible | + | 188 |
| 6 | Worker | No |  |  |  |  |  |
|  | Household Member | Yes | No | No | Susceptible | + | 182* |
|  | Household Member | Yes | No | No | Intermediate | + | 45* |
| 8 | Worker | Yes | No | Yes | Resistant | + | 398 |
|  | Worker | Yes | No | Yes | Resistant | - | 398 |
|  | Household Member | Yes | No | Yes | Susceptible | - | 30 |
| 9 | Worker | Yes | No | Yes | Susceptible | + | 30 |
|  | Household Member | No |  |  |  |  |  |
| 10 | Worker | Yes | No | Yes | Resistant | - | 398 |
|  | Household Member | Yes | No | No | Susceptible | + | 45* |
|  | Household Member | No |  |  |  |  |  |
| 11 | Worker | No |  |  |  |  |  |
|  | Worker | No |  |  |  |  |  |
|  | Household Member | Yes | No | Yes | Susceptible | - | 30 |
| 12 | Worker | Yes | No | No | Resistant | + | 1776 |
|  | Worker | No |  |  |  |  |  |
| 13 | Worker | Yes | No | No | Resistant | + | 20 |
|  | Worker | Yes | No | No | Resistant | + | 15 |
|  | Household Member | Yes | No | No | Susceptible | + | 45 |
| 16 | Worker | Yes | No | Yes | Resistant | - | 398 |
|  | Worker | Yes | No | No | Susceptible | + | 182* |
| 18 | Worker | Yes | No | No | Susceptible | + | 97 |
|  | Household Member | No |  |  |  |  |  |
|  | Household Member | No |  |  |  |  |  |

| b) AFLO participants by household. | | | | | | | |
| --- | --- | --- | --- | --- | --- | --- | --- |
| **Household** | **Participant Type** | ***S. aureus*** | **MRSA** | **MDRSA** | **Tetracycline-resistance** | ***scn*** | **CC**^b^ |
| 4 | Worker | Yes | No | No | Susceptible | + | 87 |
|  | Worker | Yes | No | No | Susceptible | + | 59 |
| 5 | Worker | Yes | Yes | No | Susceptible | + | 840 |
|  | Worker | Yes | No | No | Susceptible | + | 5 |
| 6 | Worker | Yes | No | No | Susceptible | + | 8 |
|  | Household Member | Yes | No | No | Susceptible | + | 45 |
|  | Household Member | No |  |  |  |  |  |
| 11 | Worker | Yes | No | No | Susceptible | + | 182* |
|  | Worker | No |  |  |  |  |  |
|  | Household Member | Yes | No | No | Susceptible | + | 5 |
| 12 | Worker | Yes | No | No | Susceptible | + | 30 |
|  | Household Member | No |  |  |  |  |  |
|  | Household Member | No |  |  |  |  |  |
| 13 | Worker | Yes | No | Yes | Susceptible | + | 30 |
|  | Worker | No |  |  |  |  |  |
| 15 | Worker | Yes | No | Yes | Susceptible | + | 72 |
|  | Worker | No |  |  |  |  |  |
| 17 | Worker | Yes | No | Yes | Intermediate | + | 5 |
|  | Worker | Yes | No | No | Susceptible | + | 5 |
|  | Worker | Yes | No | No | Susceptible | - | 30 |
| 18 | Worker | Yes | No | Yes | Susceptible | + | 30 |
|  | Household Member | Yes | No | No | Susceptible | + | 182 |
|  | Household Member | No |  |  |  |  |  |
| 19 | Worker | Yes | No | No | Susceptible | + | 8 |
|  | Worker | No |  |  |  |  |  |
| 20 | Worker | Yes | Yes | No | Susceptible | + | 5 |
|  | Worker | Yes | Yes | No | Susceptible | + | 5 |
| 21 | Worker | Yes | No | Yes | Susceptible | - | 30 |
|  | Household Member | Yes | No | No | Susceptible | + | 30 |
|  | Household Member | Yes | No | No | Susceptible | + | 30 |
| 22 | Worker | Yes | No | No | Susceptible | + | 45 |
|  | Household Member | Yes | No | No | Susceptible | + | 45 |
|  | Household Member | Yes | No | No | Susceptible | + | 1 |

ILO = industrial livestock operation

AFLO = antibiotic-free livestock operation

CC = clonal complex

*Novel sequence type
